# Leukocyte Telomere Length and Chronic Conditions in Older Women of Northeast Brazil: A Cross-Sectional Study

**DOI:** 10.3390/cells7110193

**Published:** 2018-11-02

**Authors:** Bruna Silva Oliveira, Catherine M. Pirkle, Maria Victoria Zunzunegui, Silvia Regina Batistuzzo de Medeiros, Ronaldo Luis Thomasini, Ricardo Oliveira Guerra

**Affiliations:** 1 Departamento de Fisioterapia, Universidade Federal do Rio Grande do Norte, Natal, Rio Grande do Norte 59078970, Brazil; 2 Office of Public Health Studies, University of Hawaii at Manoa, Honolulu, HI 96822, USA; cmpirkle@hawaii.edu; 3 Département de Médecine sociale et préventive, École de santé publique, Université de Montréal, C.P. 6128, succ. Centre-Ville, Montréal, QC H3C 3J7, Canada; maria.victoria.zunzunegui@umontreal.ca; 4 Departamento de Biologia Celular e Genética, Universidade Federal do Rio Grande do Norte, Natal, Rio Grande do Norte 59078970, Brazil; sbatistu@cb.ufrn.br; 5 Programa Multicêntrico de Pós-graduação em Ciências Fisiológicas (PMPGCF), Núcleo de Estudos de Patologias Inflamatórias e Infecciosas (NEPii) and Faculdade de Medicina de Diamantina—FAMED, Universidade Federal dos Vales do Jequitinhonha e Mucuri, Diamantina, Minas Gerais 39100000, Brazil; ronaldothomasini@gmail.com; 6 Campus Universitário Natal, Departamento de Fisioterapia, Universidade Federal do Rio Grande do Norte, Natal, Rio Grande do Norte 59078970, Brazil; roguerra@ufrnet.br

**Keywords:** telomeres, women, aging, cardiovascular risk, chronic diseases, inflammation

## Abstract

This study assessed whether telomere length is related to chronic conditions, cardiovascular risk factors, and inflammation in women aged 65 to 74 from Northeast Brazil. Participants were selected from two sources, a representative sample of the International Mobility in Aging Study (*n* = 57) and a convenience sample (*n* = 49) recruited at senior centers. Leukocyte telomere length was measured by quantitative polymerase chain reaction from blood samples in 83 women. Natural log-transformed telomere/single copy gene ratio was used as the dependent variable in the analysis. Blood analyses included inflammatory markers (high-sensitivity C-reactive protein and interleukin-6), total, low-density lipoprotein and high-density lipoprotein cholesterol, triglycerides, glucose and glycosylated hemoglobin. Self-rated health, chronic conditions, cardiovascular risk factors and inflammatory markers were not associated with telomere length. No significant independent association was found between telomere length and anthropometric measures or blood markers, even after adjusting for age, education and adverse childhood events among these older women in Northeast Brazil. Our results did not confirm the hypothesis that chronic conditions, cardiovascular risk factors or inflammation are associated with shorter telomere length in these women who have exceptional survival relative to the life expectancy of their birth cohort.

## 1. Introduction

Telomeres are the protective caps at the end of chromosomes, and have been proposed as hallmarks of aging because many in vitro and in vivo experiments indicate that telomere length (TL) is affected by cellular replication and oxidative stress, two processes acting on cellular senescence [1]. It is also possible that short telomeres induce cell senescence, which may partially regulate inflammatory processes and aging. Whether telomeres are biomarkers of aging or of overall health in old age is the subject of much research and debate [2,3].

Studies in general human populations examining associations between TL and age, sex, chronic stress of social origin, health behaviors and chronic diseases yield mixed results [2,4]. Consistent findings are age-related TL shortening and longer TL in women than men [2]. Using a life course approach, we have previously reported that three types of chronic social stress (poverty, violence and caregiving of a dependent person) are associated with telomere erosion [5]. Lower socioeconomic status (SES) is generally associated with shorter telomeres [6,7], but results are not consistent across ethnic groups [7], or in older adults who have exceeded their life expectancy at birth and who can be considered survivors of their birth cohorts [8]. 

Researchers have reported associations between short TL and increased risk of stroke [9], ischemic heart disease [10], diabetes [11], and hypertension [12]. A recent systematic review concluded that stroke, myocardial infarction, and type 2 diabetes mellitus are associated with shorter telomeres [13]. In fact, TL at the time of blood collection in cross-sectional surveys of older adults may not be related to current levels of biomarkers or health indicators, since current TL may reflect lifetime stress processes (social stress, oxidative stress, infections and subsequent inflammation). In other words, these lifetime stress processes may act on both TL and chronic conditions, accounting for the associations observed in studies. For example, no association was observed between cardiovascular risk factors (behavioral, anthropometric and blood biomarkers of risk) and leukocyte TL or short TL load in a middle-aged (40 to 54 years) and an urban middle-class sample of Spanish workers free from established cardiovascular disease [14]. Furthermore, in another middle-aged population free of cardiovascular disease at baseline, no heart structural or functional abnormalities were related to TL [15]. Poor health behaviors, in particular tobacco smoking and physical inactivity, have shown inconsistent associations with TL [16,17]. The above evidence, although conflicting, suggests stronger associations between TL and established cardiovascular, cerebrovascular and metabolic pathologies, even after adjustment for age, and weaker or non-existent associations between cardiovascular risk factors in younger populations free of cardiovascular diseases. 

A life course approach has been advocated to study TL shortening in human populations [2,5]. The associations between TL and mortality are strong in young populations and weaker in older populations [18]. In the oldest group, often defined as being over 85 years of age, the association between TL and mortality is weak and non-significant [19]. A study in Japan of the very old (aged 85–99), centenarians (100–104) and supercentenarians (105 and over) suggested that long telomeres are a prerequisite for exceptional lifespan in human aging. Those who have lived the longest appear to be precisely those able to maintain the average length of their telomeres [20].

The study of populations with high longevity (centenarians) or exceptional survival (those who reach old age in hostile environments) could be useful. We have recently conducted a study with Northeast Brazilian women who have reached old age, even though their life expectancy at birth was 33.5 years (Brazilian Institute of Geography and Statistics—IBGE: life expectancy of women born in 1940 in Rio Grande do Norte). Our study population comprised survivors of their birth cohort, meaning women who survived more than 30 years beyond their life expectancy at birth. Among these women, adverse childhood experience and low education were positively associated with TL [8]. Among these survivors, those who experienced adverse events and survived had the longest telomeres, so we concluded that their long average TL had conferred the highest survival advantage to them, despite adversity. 

Given the conflicting evidence on chronic conditions and TL, this exploratory study was designed to assess whether TL is associated with risky behaviors, chronic conditions, cardiovascular risk factors and inflammatory biomarkers in a population of women from Northeast Brazil, a region with substantial socioeconomic inequalities. We hypothesized that those with the poorest health, higher levels of cardiovascular risk factors, and/or inflammation would have shorter overall leukocyte TL. 

## 2. Materials and Methods

### 2.1. Population and Sample

This observational, cross-sectional study was carried out on a sample of community-dwelling older women (age range 65 to 74 years) of diverse socioeconomic status who are residents of the Municipality of Natal, Rio Grande do Norte, Brazil. 

The study rationale and design have been published [8]. However, some contextual information about the setting is useful for understanding the sampling strategy and observed findings. Due to a history of social inequality, education was only accessible to a very small proportion of the population in Brazil until recent decades. In Natal, the 2010 census showed that only 7.6% of women over 60 years of age had completed high school. Yet, some studies have shown that TL varies by educational attainment [8,21,22], which is a relatively stable indicator of socioeconomic status across the life course. Thus, in order to capture a wide range of education levels, we stratified our study sample by education using two sampling methods. First, we used a random sample of women registered at community health centers in five neighborhoods of Natal (*n* = 57); this sample comes from the second wave of the International Mobility in Aging Study (IMIAS) [23]. Since 92% of these IMIAS women had low education (coinciding with 2010 Brazilian Institute of Geography and Statistics census data [24]), a convenience sample to recruit older women with high education was required to implement our study design. Thus, between July and December 2014, potential participants were directly approached at senior citizen social groups and via the association of retired employees of the Universidade Federal do Rio Grande do Norte at Natal to compose the high-education group (*n* = 49). By adding this better-educated group to our sample, we were able to obtain a more heterogeneous sample of older adults for these analyses. 

Participants who had cancer (*n* = 4) were excluded because these specific conditions may have strong effects on telomere biology [25,26]. The total sample size of women in the study is 106; however, 23 participants were excluded in the analysis stage because of insufficient DNA (*n* = 17), or due to high coefficient variance or outlier values of TL measurements (*n* = 6). This resulted in a final sample size of 83.

### 2.2. Ethical Considerations 

This study was approved by the Ethics and Research Committee of Onofre Lopes University Hospital (approval number 623/11), and written informed consent was obtained from all participants. 

### 2.3. Data Collection

Data were collected during two home visits in which trained interviewers employed standardized protocols. A single interviewer visited each participant and that interviewer was responsible for all questionnaire data collection, while a laboratory professional collected peripheral blood by venipuncture during a second visit. This was done within a week of the first interview.

### 2.4. Chronic Conditions

Chronic conditions were self-reported based on the answer to the question: “Has a doctor told you that you have…?”, and including high blood pressure, diabetes mellitus, respiratory disease, coronary heart disease, cerebrovascular accident, rheumatoid arthritis, and/or osteoporosis. A count of diagnosed chronic conditions was constructed (a maximum of seven for those conditions). 

Self-reported health was assessed by the following question: “Would you say your health is excellent, very good, fair, poor or very poor?” and the responses were recoded into two categories: good if excellent/very good, and poor if fair/poor/very poor. This question has been shown to be a valid indicator of health status [27,28] and physical function in the IMIAS study, which includes a site in Natal [29]. 

The Center for Epidemiologic Study Depression Scale (CES-D) [30] has been validated in Brazilian Portuguese [31] and we used this scale to assess depression symptoms. A cutoff point of 16 [30] was used as an indicator of clinically relevant depression. 

### 2.5. Anthropometric Measures and Blood Pressure

Height was measured to the nearest 0.5 cm using Sanny^®^ stadiometer (Personal Portable Caprice, Fortaleza, Brazil), and weight was measured with a scale (Felizona, serie 3.134). Body mass index (BMI) was calculated by dividing weight in kilograms by height in meters squared. Waist circumference was measured to the nearest 0.1 cm at the umbilicus level with a fiberglass measuring tape. 

Blood pressure was measured at rest three times in the right arm using an automatic sphygmomanometer (OMRON Healthcare, Kyoto, Japan), and we used the mean of the last two values obtained.

### 2.6. Health Behaviors

Health behaviors included smoking, alcohol consumption and walking time. Participants were classified as a current smoker, former smoker, or never smoker. Alcohol consumption was determined by self-reported history of alcohol intake (never/sometimes). 

The mobility assessment tool for walking (MAT-W), a validated computer-based self-reported walking activity questionnaire that uses video animations [32] was used to assess walking time per week. Women were instructed to report the walking speed, how many days per week, and for how long per day they had walked for ‘more than 10 min without stopping during the last week’ in a usual and fast pace. We calculated the total walking duration in minutes per week as the product of the duration, and the frequency of walking.

### 2.7. Blood Collection

Blood samples were collected after overnight fasting and tested by the local laboratory service. High-sensitivity C-reactive protein (hs-CRP) was analyzed with the Roche immunoturbidimetric method. Since very high values of hs-CRP may indicate recent or current infection, for descriptive purposes we categorized hs-CRP using the following cutoff points: low (<1 mg/L), intermediate (1‒3 mg/L), high (3‒10 mg/L) and very high (≥10 mg/L) [33]. Interleukin-6 (IL-6) was measured using enzyme-linked immunosorbent assay. The results of the IL-6 plasma levels are presented in pg/mL. 

Additionally, the fasting blood analyses included the following tests: glucose (mg/dL), triglycerides (mg/dL), total cholesterol (mg/dL), high-density lipoprotein cholesterol (HDL cholesterol) (mg/dL), low-density lipoprotein cholesterol (LDL cholesterol) (mg/dL), and whole-blood glycosylated hemoglobin (HbA_1c_) in percentage.

### 2.8. Telomere Length Measurement

Genomic DNA was extracted from whole-blood leukocytes using QiAmp^®^ DNA blood isolation kits (Qiagen, Hilden, Germany) according to the manufacturer’s instructions and stored frozen in aliquots until experimentation. We determined the relative measure of TL with a quantitative polymerase chain reaction (qPCR) telomere assay as described by Cawthon [34], with some modifications described elsewhere [8].

All the samples were analyzed using the StepOne Real-Time PCR machine (Applied Biosystems—Thermo Fisher Scientific, Waltham, MA, USA) in duplicate with negative and positive controls, and standard curves. Next, four serial dilutions of human DNA (10-fold) were used as reference DNA to create the standard curve in each reaction batch for assay validation. The standard curve demonstrated linearity, with *R*^2^ = 0.99 for telomeres and beta-globin gene. The PCR efficiency ranges for beta-globin amplifications were 82‒88%, and 84‒108% for telomeres. The qPCR amplification efficiency for telomeres can exceed 100% due to the primers having more than one target in the telomere in which the TTAGGG sequence (target of primers) is naturally repeated several times. Consequently, more than one amplicon was generated for each qPCR cycle, different from the beta-globin gene (housekeeping gene, efficiency of amplifications 82‒88%). The intra-assay coefficient of variation was 2.7%, the inter-experimental coefficient was 1.67% for beta-globin gene, and 4.5% and 5.8% for telomeres, respectively. All samples were analyzed in duplicate. Quantitative values were obtained from the threshold cycle value, and the ratio of telomere repeat copy number (T) was estimated for a single gene copy number (S) for each sample (T/S ratio). 

### 2.9. Potential Confounders

As identified in the literature, age [2], education [21], and adverse childhood experiences [5,35] have been associated with telomere length in previous studies. Subjects completed a questionnaire detailing their age (years), and education level (years of schooling) and were then categorized into two groups: less than secondary education (less than 11 years), and secondary or more (11 years or more). The questions assessing exposure to adverse childhood events (ACEs)‒having witnessed family physical violence, experienced physical abuse by a close relative, hunger, poor economic status, parental death, parents’ divorce, parental unemployment, and parental abuse of alcohol‒have proven their validity in research on life course and physical function research [36,37]. 

### 2.10. Statistical Analysis

All analyses were carried out using Windows-based Statistical Package for the Social Sciences version 20.0 software (SPSS, Chicago, IL, USA); *p* values < 0.05 were considered statistically significant. 

Since T/S ratio measures were highly skewed (Kolmogorov‒Smirnov test for normal distribution *p* < 0.05), values were transformed. Thus, the natural logarithm of T/S ratio (ln T/S ratio) was used as an alternative for outcome telomere length. 

In a descriptive analysis, the mean and standard deviation were presented for normally distributed continuous variables, and median (range or inter-quartile range) for non-normally distributed variables. Dichotomous variables were presented as percentages. The hs-CRP and IL-6 variables were dichotomized considering the 75th percentile. Differences between groups were assessed using Student’s *t*-test or analysis of variance, and chi-square tests for associations between categorical variables, or the non-parametric alternative Mann‒Whitney U test, as appropriate. The associations of the anthropometric measures and blood markers with mean ln T/S ratio were assessed with linear regression models. Independent associations of TL with these variables were assessed with linear regression models adjusted for potential confounders (age, education and ACEs). 

## 3. Results

Table 1 reports the sample characteristics. Mean ln T/S ratio was significantly longer in the low-education group than in the high-education group (2.79 ± 0.9 compared with 2.03 ± 0.9, *p* = 0.0001). Women in the low-education group were significantly older (*p* = 0.0001) and reported more ACEs (*p* = 0.02) compared to the secondary or higher education group. The women with lower education reported poorer health, a greater overall number of chronic diseases and more osteoporosis. Nearly 25% of those in the low-education group had CES-D scores of 16 or greater. However, there were no statistically significant differences for any of the remaining chronic conditions (hypertension, stroke, chronic heart disease, chronic lung disease, and rheumatologic conditions), nor diastolic blood pressure. Systolic blood pressure was significantly higher in the low-education group. Since there were too few stroke outcomes to examine, this condition was not included in subsequent analyses.

Glucose mean values were highest in the low-education group (*p* = 0.002). No significant differences between the two groups were observed according to lipids and inflammatory blood markers (total cholesterol, HDL cholesterol, LDL cholesterol, triglyceride, HbA_1C_, hs-CRP or IL-6). Regarding anthropometric measures, the differences were statistically significant only for height and waist circumference; there were no statistically significant differences in body mass index or weight. Finally, almost three-fourths of the low-education group reported never having drunk alcohol (71.4%) compared to 39% in the higher education group. There was no difference in total walking time per week between the groups, nor in reported smoking habits.

Table 2 summarizes the results of adjusted and unadjusted analyses of the ln T/S ratio for chronic conditions, health behaviors and blood biomarkers. While not statistically significant, longer mean ln T/S ratios were observed for nearly all of those who reported chronic conditions, and an unadjusted association of longer telomeres with depression was observed (*p* = 0.03). This association was attenuated and lost statistical significance after adjustment for the potential confounders of age, education and ACEs. No statistically significant associations were observed with measures of inflammation. No statistically significant association was observed between TL and the remaining potential correlates after adjusting for age, education and ACEs. Unadjusted and adjusted associations between ln T/S ratio and anthropometric measures and blood markers are shown in Table 3. No statistically significant associations were observed. Exploratory analyses of these associations by educational group we did not observe any meaningful differences in the results (data not shown).

## 4. Discussion

Research on the associations between chronic conditions and TL has produced inconclusive results [11,14,38]. Moreover, these associations are strongly confounded by age: they tend to be significant in studies with a wide range of ages and to be weaker or non-significant in studies of older adults [2]. There is evidence that confounding factors such as life course adversity and educational attainment [5,8] may influence both TL and risk of chronic conditions, thus accounting for the associations observed in some cross-sectional studies. Notably, no statistically significant associations between TL length and chronic conditions, self-rated health, health behaviors or blood biomarkers were observed in our sample of women, who have lived long enough to double their life expectancy at birth after adjustment by the strong confounders of age, education and ACEs.

Our results are similar to those obtained among centenarians in Japan, a population with exceptional survival, where TL length was unrelated to cognition, capability assessed by Barthel index and number of chronic conditions; the association between inflammation (the strongest predictor of survival) and TL was reversed in centenarians and lost in supercentenarians [20]. Appleby, et al. [39] examined associations between health status and TL in a sample of adults aged 49–51 but did not find any associations, even adjusting for gender, ethnicity and socioeconomic status. In addition, studies conducted in middle-aged populations free of established cardiovascular disease have found no association between TL and cardiovascular risk factors (BMI, waist circumference, glucose, hypertension, years smoking, total cholesterol, triglycerides, glycosylated hemoglobin and alcohol intake) [14,15], but those studies were conducted in an age group in which the effects of cardiovascular risk factors may not have had enough time to influence TL, if indeed there is an association.

We have observed that TL is likely not associated with health behaviors or health indicators in populations that have been able to maintain relatively long telomeres, have survived to exceptionally old ages (centenarian study) or have been shown to have exceptional survival relative to the life expectancy of their birth cohort (older women from Northeast Brazil). These selected populations may be protected from TL-dependent cell-senescence and may have remained protected from common pathologies or postponed chronic inflammation. In other words, TL may not be associated with health status in these populations because the “fittest women” survived to older ages. The unexpected results in our low-education sample, e.g., greater mean ln T/S ratio despite poorer overall health, may reflect a type of selective survival. Numerous studies document that, as disparate groups get older, gaps in health outcomes between the best and worst-off narrow [40,41,42]. Some attribute the shrinking gap to the *aging-as-leveler* hypothesis, which posits that aging involves negative consequences to both the advantaged and disadvantaged, and that those with advantaged earlier lives have the most to lose. Others argue that selective survival of the most biologically “resilient” may explain shrinking health disparities in older populations [40]. The latter explanation may be of particular relevance to our observations in this cohort from Northeast Brazil.

Furthermore, as suggested by Arai, Martin-Ruiz, Takayama, Abe, Takebayashi, Koyasu, Suematsu, Hirose and von Zglinicki [20], the high levels of inflammation observed in centenarians may be a relatively late “catch-up” event. If this is true, the centenarians in Japan who may have survived with longer telomeres than expected for their age could be experiencing an increase in inflammation, which may have manifested earlier in members of their birth cohort who did not survive to old age. This is also observed in our study of Brazilian older women who have higher rates of inflammation (IL-6 was 2.00 pg/mL in total sample) compared to what has been published in studies of older adults with similar ages in high-income countries, such as in Ferrucci, et al. [43] (mean IL-6 was 1.1 pg/mL in women 65–74 years old). However, IL-6 is a multifunctional glycosylated protein [44] and physical activity and nutrition influence its response [45]. Additionally, since TL biology changes during the life course, the direct study of pro- and anti-inflammatory cytokine balance and TL in older adults poses many research challenges, and longitudinal population studies on this relationship are not achievable at this time.

Alternative explanations for our finding of no association between health status and TL in our sample of older women from Northeast Brazil need to be considered. First, the limitations of the present study are related to the great variability of TL in humans. Since our multivariate models show small beta coefficients for most of the independent variables examined, it is questionable whether associations would be meaningful, even if we had greater statistical power through a larger sample size. There is a possibility of random errors due to the one-time measurement of TL that might lead to underestimating the associations between TL and health outcomes. In addition, we lack an absolute measure of TL, and we were unable to use the conventional cutoff point of leukocyte TL < 3 kilobases to characterize short telomere load [14,46]. Furthermore, most of the literature on TL and aging is based on average TL measures. More work examining short TL burden is needed in low- and middle-income settings such as Northeast Brazil. Although we also included self-reported conditions and these questions could be subject to measurement error, previous studies have assessed the reliability and specificity of self-reported chronic conditions [47,48,49] and, overall, self-reporting is valid. This is the first study in older women from Northeast Brazil to show that whole-blood leukocyte TL is not associated with health status. Since TL biology is complex, research among older adults would benefit from international studies including populations with different life expectancies, thus reflecting the variability in life course exposures and outcomes.

## Figures and Tables

**Table 1:** Study population characteristics.

Characteristics	Total Sample, *n* = 83	Less Than Secondary Education, *n* = 42	Secondary or More Education, *n* = 41	*p*
Age, years, median (min–max)	69 (65–74)	69 (66–74)	67 (65–74)	0.0001
T/S ratio, median (IQR)	0.86 (0.3)	1.02 (0.4)	0.64 (0.6)	0.0001
ln T/S ratio, mean ± SD	2.4 ± 0.9	2.8 ± 0.9	2.0 ± 0.9	0.0010
Adverse childhood experiences				0.0200
None, %	62.7	50	75.6	
1 or more, %	37.3	50	24.4	
**Chronic conditions**				
Number of chronic conditions, %				0.0100
None or 1	24.1	14.3	34.1	
2	38.6	38.1	3.9	
3	24.1	23.8	24.4	
4 or more	13.3	23.8	2.4	
Hypertension	67.5	69.0	65.9	0.7600
Diabetes	36.1	45.2	26.8	0.0800
Stroke	2.4	2.4	2.4	0.9900
Cardiovascular disease	8.4	7.1	9.8	0.6700
Chronic lung disease	10.8	16.7	4.9	0.1600
Arthritis, rheumatism or osteoarthritis	67.5	71.4	63.4	0.4400
Osteoporosis	32.5	47.6	17.1	0.0030
Systolic blood pressure, mmHg, mean ± SD	136.8 ± 17.6	141.0 ± 18.3	132.4 ± 16.0	0.0200
Diastolic blood pressure, mmHg, mean ± SD	75.6 ± 10.3	75.8 ± 11.7	75.3 ± 8.9	0.8300
Self-reported health				0.0010
Good, %	37.3	11.9	88.1	
Poor, %	62.7	63.4	36.6	
Depression (CES-D)				0.0010
Score < 16, %	88	76.2	100	
Score ≥ 16, %	12	23.8		
**Blood biomarkers**				
Cholesterol total (mg/dL), mean ± SD	216.2 ± 49.5	226.2 ± 52.0	206 ± 45.1	0.0600
HDL cholesterol, mg/dL, mean ± SD (*n* = 75)	54.5 ± 12.9	53.0 ± 10.1 (*n* = 36)	55.9 ± 15.0 (*n* = 39)	0.3400
LDL cholesterol, mg/dL, mean ± SD (*n* = 75)	117.8 ± 37.1	114.0 ± 35.5 (*n* = 36)	121.3 ± 38.7 (*n* = 39)	0.4000
Triglycerides, mg/dL, median (min–max) (*n* = 75)	121.3 (43.4–369.1)	125.8 (43.4–252.5) (*n* = 36)	118 (51.4–369.1) (*n* = 39)	0.5300
Glucose, mg/dL, median (min-max) (*n* = 82)	99.8 (62–456)	110 (62–456) (*n* = 41)	89.3 (65.4–169) (*n* = 41)	0.0020
HbA_1C_, %, median (min–max)	5.9 (1.0–9.8)	5.0 (4.9–14.4)	5.9 (5.2–8.5)	0.0900
hs-CRP, %				0.2500
Low (<1 mg/L)	31.3	26.2	36.6	
Moderate (1–3 mg/L)	25.3	21.4	29.3	
High (3–10 mg/L)	32.5	35.7	29.3	
Very high (≥10 mg/L)	10.8	16.7	4.9	
IL-6 (pg/mL), median (min–max)	2.0 (1.0–9.8)	2.2 (2.0–9.8)	2.0 (1.0–7.3)	0.1100
**Anthropometric measures**				
Weight, kg, mean ± SD	67.7 ± 14.2	67.1 ± 13.9	68.6 ± 14.6	0.6400
Height, meters, mean ± SD	152.4 ± 5.8	150.8 ± 4.8	154.0 ± 6.3	0.0100
Body mass index, Kg/m^2^, mean ± SD	29.2 ± 5.8	29.4 ± 5.6	28.9 ± 6.0	0.6800
Waist circumference, cm, mean ± SD	96.5 ± 13	99.9 ± 13.4	92.9 ± 11.8	0.0100
**Health behaviors**				
Smoking status				0.5500
Never smoker	62.7	59.5	65.9	
Former smoker	36.1	38.1	34.1	
Current smoker	1.2	2.4		
Never drank alcohol	55.4	71.4	39	0.0300
Minutes of walking per week, median IQR	50 (120)	40 (80)	60 (170)	0.1800

Note. ln T/S ratio = logarithm mean relative ratio telomere/single copy gene; IQR = inter-quartile range; HDL = high-density lipoprotein; LDL = low-density lipoprotein; HbA_1C_ = glycosylated hemoglobin; hs-CRP = High-sensitivity C-reactive protein; IL-6 = Interleukin-6. p value for analysis of variance, *t* Test or Chi-square test, or Mann‒Whitney U test.

**Table 2:** Mean (unadjusted and adjusted) of ln T/S ratio for chronic conditions, health behaviors and inflammatory markers (*n* = 83).

Variables	Unadjusted		Adjusted ^a^	
	Mean (95% CI)	*p* Value	Mean (95% CI)	*p* Value
**Chronic Conditions**				
Hypertension		1.00		0.88
No	2.42 (2.05–2.79)		2.45 (2.10–2.79)	
Yes	2.41 (2.16–2.67)		2.72 (2.18–2.66)	
Diabetes		0.24		0.47
No	2.32 (2.06–2.58)		2.37 (2.13–2.62)	
Yes	2.58 (2.23–2.93)		2.52 (2.19–2.85)	
Cardiovascular disease		0.49		0.43
No	2.39 (2.17–2.61)		2.40 (2.19–2.61)	
Yes	2.66 (1.93–3.38)		2.68 (2.0–3.34)	
Chronic lung disease		0.73		0.29
No	2.35 (2.17–2.61)		2.39 (2.18–2.60)	
Yes	2.66 (1.93–3.38)		2.72 (2.13–3.31)	
Arthritis, Rheumatism or Osteoarthritis		0.75		0.94
No	2.37 (2.00–2.74)		2.41 (2.07–2.76)	
Yes	2.44 (2.18–2.70)		2.43 (2.19–2.67)	
Osteoporosis		0.16		0.69
No	2.31 (2.06–2.57)		2.40 (2.16–2.64)	
Yes	2.63 (2.27–3.00)		2.48 (2.13–2.85)	
Self-reported health		0.06		0.91
Good	2.16 (1.82–2.49)		2.44 (2.07–2.82)	
Poor	2.57 (2.31–2.83)		2.41 (2.16–2.68)	
Depression (CES-D)		0.03		0.28
Score < 16	2.33 (2.11–2.55)		2.38 (2.17–2.60)	
Score ≥ 16	3.05 (2.46–3.64)		2.73 (2.14–3.31)	
**Health behaviors**				
Smoking		0.43		0.55
Never	2.35 (2.08–2.62)		2.48 (2.25–2.71)	
Current smoker or former smoker	2.52 (2.18–2.87)		2.26 (1.83–2.69)	
Alcohol		0.34		0.39
Never	2.53 (2.26–2.79)		2.44 (2.19–2.71)	
Sometimes	2.24 (1.90–2.58)		2.43 (2.06–2.72)	
**Inflammatory biomarkers**				
hs-CRP		0.13		0.44
Low (<1 mg/L)	2.21 (1.85–2.58)		2.34 (1.99–2.70)	
Moderate (1–3 mg/L)	2.39 (1.98–2.80)		2.46 (2.07–2.84)	
High (3–10 mg/L)	2.40 (2.04–2.76)		2.34 (2.00–2.68)	
Very high (≥10 mg/L)	3.09 (2.47–3.72)		2.87 (2.27–3.47)	
IL-6		0.19		0.39
Lower quartile (<75th)	2.34 (2.10–2.58)		2.38 (2.15–2.61)	
Higher quartile (75th and over)	2.66 (2.23–3.09)		2.56 (2.17–2.95)	

Note. ^a^ Mean of ln T/S ratio adjusted for age, education and childhood adverse experience.

**Table 3:** Linear regressions of ln T/S ratio by anthropometrics measures, minutes of walking, blood metabolic and inflammatory biomarkers (*n* = 83).

	Unadjusted Model		Adjusted Model ^a^	
	B Coefficient ± SE	*p* Value	B Coefficient ± SE	*p* Value
Weight, Kg	0.001 ± 0.008	0.87	−0.030 ± 0.007	0.67
Height, meters	−0.010 ± 0.020	0.43	−0.004 ± 0.020	0.81
Body mass index, kg/m^2^	0.008 ± 0.020	0.66	−0.007 ± 0.002	0.69
Waist circumference, cm	0.010 ± 0.008	0.12	0.0001 ± 0.008	0.96
Minutes of walking per week	0.0001 ± 0.001	0.65	0.00001 ± 0.001	0.99
Total cholesterol, mg/dL	0.001 ± 0.002	0.57	0.0001 ± 0.002	0.82
HDL cholesterol, mg/dL	−0.040 ± 0.009	0.66	0.001 ± 0.008	0.90
LDL cholesterol, %, mg/dL	0.002 ± 0.003	0.47	0.003 ± 0.003	0.26
Triglycerides, mg/dL	0.001 ± 0.002	0.45	0.001 ± 0.002	0.67
Glucose, mg/dL	0.001 ± 0.002	0.58	0.0001 ± 0.002	0.83
HbA_1C_, %	0.180 ± 0.210	0.40	0.180 ± 0.200	0.36
hs-CRP, mg/L	0.030 ± 0.020	0.06	0.020 ± 0.020	0.16
IL-6, pg/mL	0.070 ± 0.070	0.32	0.020 ± 0.060	0.69

Note. ^a^ Adjusted by age, education and adverse childhood events.
